# Pharmaceutical digital marketing and governance: illicit actors and challenges to global patient safety and public health

**DOI:** 10.1186/1744-8603-9-45

**Published:** 2013-10-16

**Authors:** Tim K Mackey, Bryan A Liang

**Affiliations:** 1 Institute of Health Law Studies, California Western School of Law, 350 Cedar Street, San Diego, CA 92101, USA; 2 San Diego Center for Patient Safety, University of California San Diego School of Medicine, 200 W. Arbor Drive, San Diego, CA 92103-8770, USA; 3 Department of Anesthesiology, University of California San Diego School of Medicine, 200 W. Arbor Drive, San Diego, CA 92103-8770, USA

**Keywords:** Internet governance, Online pharmacies, Direct-to-consumer advertising, Global health, Social media, Counterfeit medicines, Cybercrime

## Abstract

**Background:**

Digital forms of direct-to-consumer pharmaceutical marketing (eDTCA) have globalized in an era of free and open information exchange. Yet, the unregulated expansion of eDTCA has resulted in unaddressed global public health threats. Specifically, illicit online pharmacies are engaged in the sale of purportedly safe, legitimate product that may in fact be counterfeit or substandard. These cybercriminal actors exploit available eDTCA mediums over the Internet to market their suspect products globally. Despite these risks, a detailed assessment of the public health, patient safety, and cybersecurity threats and governance mechanisms to address them has not been conducted.

**Discussion:**

Illicit online pharmacies represent a significant global public health and patient safety risk. Existing governance mechanisms are insufficient and include lack of adequate adoption in national regulation, ineffective voluntary governance mechanisms, and uneven global law enforcement efforts that have allowed proliferation of these cybercriminals on the web. In order to effectively address this multistakeholder threat, inclusive global governance strategies that engage the information technology, law enforcement and public health sectors should be established.

**Summary:**

Effective global “eHealth Governance” focused on cybercrime is needed in order to effectively combat illicit online pharmacies. This includes building upon existing Internet governance structures and coordinating partnership between the UN Office of Drugs and Crime that leads the global fight against transnational organized crime and the Internet Governance Forum that is shaping the future of Internet governance. Through a UNODC-IGF governance mechanism, investigation, detection and coordination of activities against illicit online pharmacies and their misuse of eDTCA can commence.

## Background

Health-related technologies are undergoing an evolution driven by the rapid emergence and dominance of the Internet in everyday life. According to the International Telecommunications Union (“ITU”), an estimated 2.7 billion people (39% of the world’s population) are online in 2013 [1]. These online users are increasingly becoming health information seekers and consumers. A recent Pew Internet survey found that 72% of USA adult online users search for health and medical information online and approximately 1/3^rd^ engage in self-diagnosing of their health problems [2]. This trend is not solely limited to the USA, with recent surveys indicating one in two Internet users, in a diverse collection of 12 different countries, also engage in self-diagnosing [3].

This growth has led to the development of new concepts in health, including “e-Health”, i.e., a multidisciplinary “intersection of medical informatics, public health and business, referring to health services and information delivered or enhanced through the Internet and related technologies [4].” Similarly, the concept of “Medicine 2.0” is used to describe interactive social network and consumer-directed use of health-related applications, services, and tools [5]. e-Health developments have highlighted the benefits of these technologies with an emphasis on their potential to improve health education, outreach, disease surveillance, collaboration, communication between patients and providers, and support of clinical decision-making [5-11]. In turn, these benefits can result in improved access and delivery of healthcare services (including in low-income and rural settings), reduced associated healthcare costs, and better health outcomes through technology investment [5-11]. Consequentially, although challenges remain for its full potential to be realized, e-Health technologies are expanding in global adoption [7,9,12].

Yet, e-Health advances also enable health-related digital marketing and promotion that can be of questionable quality, reliability, origin and authenticity [13-15]. General advocacy for free and open information exchange that is self-governed has led to lack of adequate Internet governance. This consequently has given rise to the globalization of pharmaceutical marketing and forms of digital direct-to-consumer advertising (“eDTCA”) as they evolve with Internet technologies [16,17]. However, it is important to note that direct-to-consumer advertising (“DTCA”) is only legally permitted in the USA and New Zealand among developed countries, yet DTCA and eDTCA transmission via the Internet and other mediums has been shown to cross geopolitical borders and pose unaddressed regulatory and public health problems [17,18]. Though online health information has potential utility if properly filtered, vetted, and framed within eHealth literacy needs, studies have demonstrated that quality of online health information (including pharmaceutical marketing) can be highly uneven as it is largely unregulated [15].

Perhaps most disturbing, vulnerabilities in the current global Internet governance and the pharmaceutical promotion regulatory regime provides fertile ground for promotion of dangerous medicines by illicit, or “rogue” online pharmacies [19,20]. Illicit online pharmacies market the sale of purportedly safe, legitimate product that may in fact be counterfeit or of substandard quality [15,19-21]. This activity should not be conflated with the global debate over the appropriate definition of “counterfeit” medicines, which is currently mired in considerations involving intellectual property rights and access to medicines [22]. For purposes of clarity, we adopt the general standard that counterfeit medicines consist of those (a) deliberately produced with substandard quality; (b) those fraudulently labeled with respect to their identity/origin; or (c) that are otherwise tainted, adulterated, or made ineffective or harmful [22].

Illicit cyberpharmacies that sell questionable medications without a prescription hence, represent a form of cybercrime that has been described as the preeminent global governance challenge of the 21^st^ century [23]. In fact, illicit online pharmacies represent a *dual* threat in that they present both challenges to global public health and risks to global cybersecurity that remain largely unaddressed by international stakeholders [19,20]. Enabling this digital trade are online criminal actors that exploit eDTCA mediums intended for use by legal actors (but possibly illegal in jurisdictions that do not permit DTCA marketing) to drive sales of their potentially dangerous services and products [13,17].

Given these ongoing public safety concerns that intersect between the global health and information technology (“IT”) policy domains, it is essential to examine the mechanisms and infrastructures utilized by illicit online pharmacies to determine needed strategies in combating this unique form of transnational cybercrime. Consequently, we first describe the potential public health risks, patient safety dangers, and cyber security issues associated with illicit online pharmacies. On this basis, we also review key efforts by a variety of actors in the international community attempting to address this issue. Finally, we propose a novel global governance approach emphasizing public health priorities in current Internet governance activities as a foundation to combat illicit online pharmacies and their exploitation of unregulated eDTCA.

## Patient safety risks of illicit online pharmacies

Several studies have explored the public health implications of industry-based legally-allowable DTCA. Possible negative consequences include dissemination of misleading or unbalanced information about the risks and benefits of medications, overutilization of expensive prescription drugs, aggressive promotion of pharmaceuticals with questionable safety profiles often at early stages in their product life-cycle, and negative patient-physician interactions [24-29].

Yet eDTCA use by illicit online pharmacies represents an even greater risk to patient safety and public health as this enterprise is largely populated by criminal actors, websites are unregulated and lack required licensure for operation, and eDTCA content often consists of misleading or fraudulent information directly targeted at the patient [13,17]. Indeed, illicit online pharmacies have been found to market a wide array of pharmaceutical products including those subject to critical shortages, vaccines, non-communicable disease medicines, essential medicines, and controlled substances to vulnerable patient populations with limited access and resources [30-34]. These illegal vendors use a variety of means to induce illicit purchases, but, as most research suggests, the majority focus on “no prescription required” approaches that represent the highest risk to consumers [13-15,19].

Harm to prospective consumers sourcing medicines from illicit no prescription online pharmacies comes in two primary forms. First, consumers may engage in self-diagnosing and self-prescribing of their health conditions without partnership of a medical professional [19]. This behavior can lead them to purchase medications that are unnecessary, have contraindications, have abuse potential or may otherwise be dangerous to their health even in instances of sourcing authentic product [19,31]. Second, even if a patient appropriately self-diagnoses their condition, purchasing from an illicit online pharmacy provides no guarantee of quality or safety and can lead to the consumption of counterfeit medicines that are substandard or otherwise dangerous [19].

In this discussion, we focus on the illegal online marketing and sale of any medication *without* a prescription, which is a clear violation of laws and regulations of the vast majority of countries requiring controls for dispensing of regulated medical products. We focus on this predominant subset of online pharmacies as the quality and safety of medications sourced from “no prescription” websites largely cannot be determined, and even if there is the possibility of sourcing authentic medication, patients may nevertheless be exposed to safety risks as previously described. Specifically, the Internet poses unique challenges to counterfeit detection in that purchasing is difficult to trace, and testing products illegally sourced by individual online consumers is inherently difficult, intrusive and costly. Hence, false and misleading marketing utilized by no prescription online pharmacies can induce the unregulated and illegal sale of medications of unknown quality, with the consumer having no way of ensuring what they are sourcing is safe [19].

These collective challenges have resulted in a general lack of data needed to identify the exact percentage of counterfeit medicines sold by illicit online pharmacies. However, documented patient injury and deaths in multiple countries directly associated with online medicines purchasing involving both substandard medications and authentic medicine taken incorrectly provides a clear indication of ongoing patient safety risks justifying regulation and enforcement [19,22]. Further, increased online self-prescribing behavior in combination with a recent USA Food and Drug Administration (“FDA”) reporting that 23% of adult Internet consumers purchased a prescription medication online (which could include both legitimate and illicit providers), provides a clear indication that additional research is needed to adequately determine the scope of this problem [35].

Despite lack of comprehensive data, organizations such as the USA National Association of Boards of Pharmacy (“NABP”) have attempted to analyze how widespread is the practice of illegal online marketing and operation of illicit online pharmacies. In March, 2013, NABP released its study of approximately 10,000 websites, reporting 97% of them did not meet adequate pharmacy laws and practice standards and 86% of these not requiring a valid prescription [36]. This most recent assessment by the NABP reveals that there has not been a reduction in the presence of illicit and “no prescription” providers [36,37]. An earlier World Health Organization (“WHO”) report also estimated that greater than 50% of websites failing to disclose their physical address are engaged in the sale of counterfeit medicines [38].

In addition, increasingly Medicine 2.0 or social media forms of eDTCA have been identified as a platform for the promotion of illicit online pharmacies [13,17]. This includes the use of popular social media sites of Facebook and Twitter that have widespread global adoption [13]. A recent study examined the vulnerabilities associated with popular Medicine 2.0 technologies, and found that illicit “no prescription” eDTCA promotion by a fictional online pharmacy was easily accessible and reached a number of global users in diverse countries, including developed countries, low-and-middle income countries (“LMICs”), as well as certain emerging “BRIC” countries (i.e., Russia and China) [17].

## Governance and cybercrime challenges of online pharmacies

Although there is potential for harm from both legal industry-based and illicit online pharmacy eDTCA, illicit online marketing activity should be prioritized in global health and Internet governance efforts. There is, for the most part, no domestic means to ensure accountability for illegal and harmful actions by these criminal actors originating across geopolitical lines [14,19]. Practically speaking, even assuming there are empowering applicable laws, online pharmacies having a physical or infrastructural presence outside of a nation’s jurisdiction may not be reachable to regulate or police, compared with legal companies that are multi-national and accountable to regulatory mandates [14,19,20,39]. In comparison, illicit online pharmacies completely bypass domestic criminal laws, national medicines regulatory systems, local law enforcement, and traditional public health access controls (e.g., protecting children and adolescents), since they are virtual, easily anonymized, and market and sell directly to consumers outside professional medical oversight [15,19,20].

The global vacuum of effective governance and regulatory approaches against illicit online pharmacies has predictably attracted large criminal networks looking to profit from this trade. Consequently, illicit online pharmacies threaten state sovereignty and global security due to their association with transnational organized crime syndicates, as well as cybercrime and cybersecurity threats [20]. For example, in one prosecuted case, the Russian Mafiya used online pharmacy distribution, massive email spam, and introduction of computer viruses to produce greater than $150 million in profits from illicit online pharmacy operations before it was brought down [40]. Yet this is a case of *successful* detection and prosecution in a digital environment where the majority of pharmaceutical crime often goes undetected. This continued expansion of pharmaceutical cybercrime is evident in the continued proliferation of illicit online pharmacies and their increasing use of various forms of eDTCA. eDTCA use includes spam and other solicitations that act as a vehicle for fraud, phishing scams, viruses, malware, and spyware, often targeted towards vulnerable consumer groups [15,19]. Indeed, close to 1/3^rd^ of spam messages are health-related, generally eDTCA originating from suspect online pharmacies [41].

## Internet pharmacy governance and enforcement efforts

A wide array of public health and law enforcement stakeholders, including WHO, the UN Office of Drugs and Crime (“UNODC”), the International Criminal Police Organization (“Interpol”), the FDA, NAPB, the USA Federal Bureau of Investigation, the International Pharmaceutical Federation, the European Federation of Pharmaceutical Industries and Associations, the Pharmaceutical Research and Manufacturers of America, the Generic Pharmaceutical Association, the Pharmaceutical Security Institute and numerous other public and private sector groups have specifically recognized the global challenges posed by the Internet and illicit online pharmacies [19,22,42-44]. Yet few solutions have emerged to confront this form of globalized pharmaceutical cybercrime.

**Figure 1:**
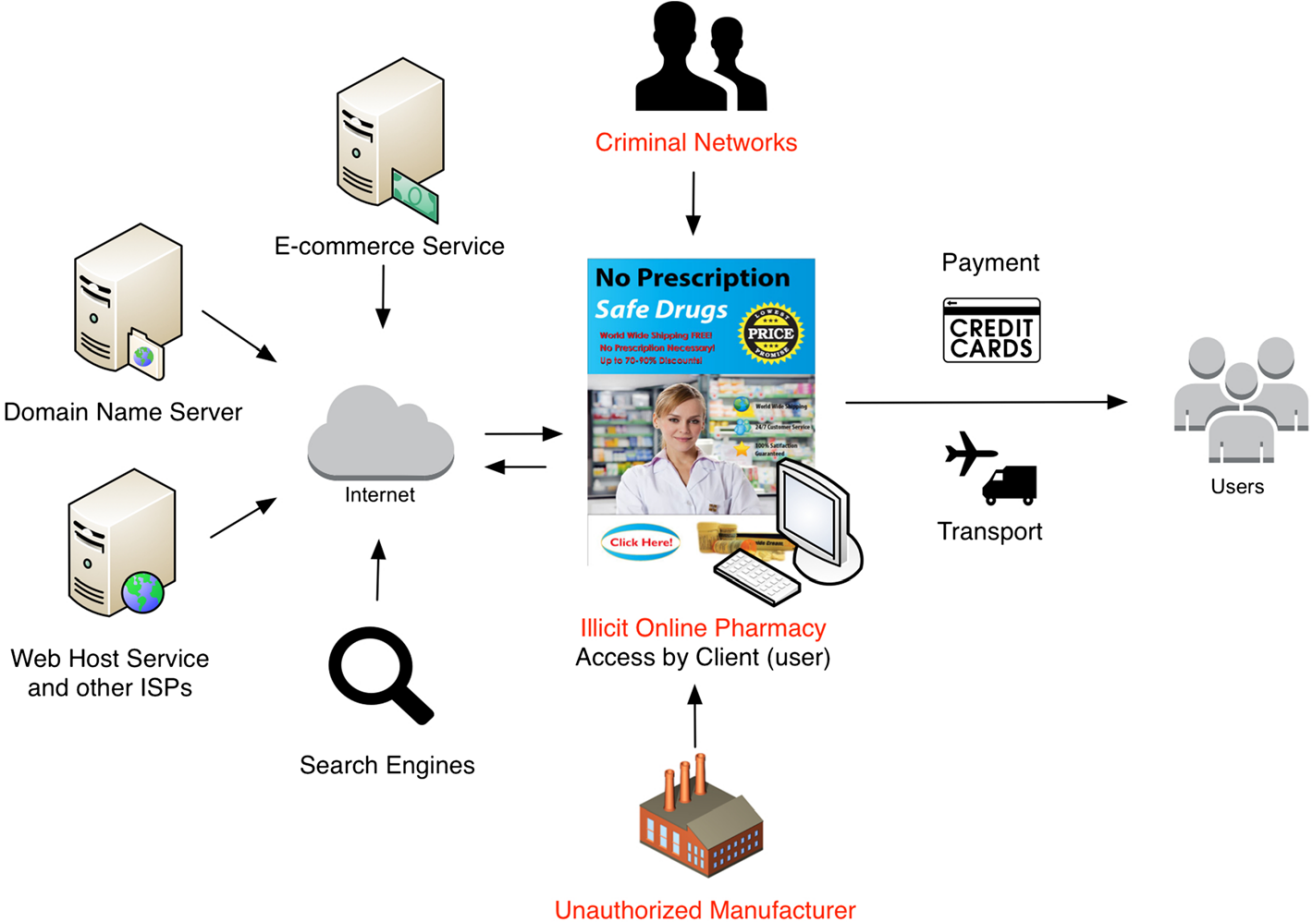
The illicit online pharmacy ecosystem.

Further, strategic approaches are complicated given the unclear applicability of domestic laws and general lack of enforcement in the Internet service sector. For example, although this criminal activity is perpetrated by a host of clearly criminal actors (e.g., illegal manufacturers, organized crime, illicit online pharmacies), others, including Internet Service Providers (e.g., search engines, social media platforms, hosting companies, payment processors, affiliate sites, transportation companies, etc.), enable illicit online pharmacy operations but often span multiple jurisdictions and legal regimes (including those that may be exempt from liability) [19-21,44] (See Figure 1). This patchwork of various illicit and legal actors makes it difficult to detect, prevent and engage in enforcement efforts against illicit online pharmacies at the domestic level, consequently requiring global coordination and cooperation that is elusive without effective multistakeholder governance [20,22].

Below we identify some of the current global governance efforts attempting to address illicit online pharmacy regulation at the national government level, voluntary governance mechanisms addressing the promotion of pharmaceuticals, and global law enforcement efforts taken against illicit online pharmacies.

### Sovereign regulatory efforts

Legislative responses from national governments to address the proliferation of illegal online pharmacies and their eDTCA have been largely absent. Though sovereign drug regulatory authorities usually regulate prescription drugs and medical products sold by traditional brick-and-mortar pharmacies, most have failed to regulate online pharmacies as a distinct category [15,19]. For example, a recent Member State survey by the WHO Global Observatory for e-Health (“GOe”) found 66% of respondents had no legislation either explicitly allowing or prohibiting Internet pharmacy operations [15]. Of those countries regulating online pharmacies, only 19% prohibited this practice, and indeed, 7% allowed it without adequate law enforcement considerations [15].

Importantly, developing countries, with fewer resources, were more likely to be silent on regulation of this public health risk [15]. Indeed, the vast majority of respondents (86%) did not regulate, accredit, or certify Internet pharmacy sites, and 75% had no regulations permitting or prohibiting the online purchasing of pharmaceuticals from other countries, a practice which has already been identified as creating significant and demonstrable health risks [15]. Even among the few countries that prohibit online medicine foreign sourcing, only 20% of this group had specific law enforcement consequences [15].

Even if a country has enacted specific legislation, such efforts may be inadequate and outdated to effectively deal with the rapidly changing pace of the Internet environment. As an example, the USA, which has a strong drug regulatory regime and extensive technological access, enacted the Ryan Haight Online Pharmacy Consumer Protections Act in 2008, regulating the online sale of controlled substances [19,44]. Yet on closer inspection, this law has significant limitations.

For example, the Act is limited in scope to only USA Drug Enforcement Administration scheduled controlled substances, which does not adequately cover the host of medication therapeutic classes currently sold illicitly over the Internet [45]. It also limits its oversight to illicit sellers located in the USA, despite the observation that the bulk of these illicit marketers and sellers are outside the country [45]. Further, there appear to be no successful prosecutions under the Act and reports by Internet monitoring companies indicate that illicit online pharmacies marketing the “no prescription” sale of controlled substances continue to operate despite passage of the law [46].

### Voluntary governance mechanisms

There have also been voluntary guidelines issued to address DTCA and other form of medicines promotion. For example, the WHO Ethical Criteria for Medicinal Drug Promotion (“WHO Criteria”) provides basic aspirational tenets [47]. While these criteria were focused on legitimate actors, principles contained therein are relevant to current illicit online pharmacy promotional activities. These guidelines indicate that DTCA medicines promotion should: (a) be reliable, accurate and truthful; and (b) not contain misleading statements or omissions that would give rise to risk [47].

Despite their seeming uncontroversial, foundational nature, decades after introduction of the WHO Criteria, WHO surveys have found a large fraction (~1/3^rd^) of countries have little to no regulatory oversight over pharmaceutical promotion [48,49]. Further, even more concerning, even fewer countries have adequate capacity or resources to regulate either licit or illicit pharmaceutical promotion [48,49], exposing their populations to public health and individual patient safety harms associated with DTCA and eDTCA.

Crucially, the WHO Criteria also fail to specifically address emerging challenges of the Internet as a medium for promotion and influencing health behavior. Further, its voluntary nature highlights certain global governance limitations in regulating marketing by illicit online pharmacies as guidelines only hold potential influence over good faith actors, not criminal actors who dominate this space. In this regard, other efforts, such as the NGO HealthOntheNetFoundation Code of Conduct recommending websites voluntarily adhere to certain principles and undergo certification to ensure quality of online health information, may also be limited in effectiveness and lack applicability to online pharmacies [15].

Other voluntary efforts are marginally better because they identify specific, legitimate online pharmacies that have undergone credentialing and necessary domestic inspection requirements. For example, credentialing agencies partnering with national drug regulators, such as NABP and the Royal Pharmaceutical Society of Great Britain, have created their own programs as well as maintain lists of websites that they have identified as highly suspect [15,19,37]. The European Parliament has also attempted to specifically address illegal Internet medicine sales through issuing Directives aimed at developing enforcement measures and differentiating illicit actors from legitimate sources by using credentialing and a common logo [50]. However, participation in these programs has been limited, and consumers have limited knowledge of the value of these programs in engaging in online sourcing behavior.

### Global law enforcement efforts

Although national online pharmacy legislation and voluntary governance initiatives attempting to regulate global pharmaceutical promotion have been limited in effectiveness, criminal law enforcement efforts have shown promise. These largely field-based operations have targeted the shut down of illicit online pharmacies and are coordinated by international organizations such as Interpol and the UNODC.

In fact, recently, Interpol, the world’s largest international police organization, announced a comprehensive multimillion-dollar global initiative to fight pharmaceutical crime in cooperation with 29 of the world’s largest pharmaceutical companies [51]. This investment in online pharmacy cybercrime prevention includes the recent “Operation Pangea VI”, a multi-stakeholder initiative involving law enforcement, the pharmaceutical and wholesaler industries, the Internet service sector, credit card companies, health regulators and customs agencies cooperating to target enforcement against illicit online drug sellers [52]. Operation Pangea VI resulted in a reported 9.8 million potentially dangerous medicines confiscated (at an estimated worth of USD$41 million), shut down of greater than 9,000 websites, and arrested or placed under investigation 58 individuals around the world [52]. This represents a marked increase in online enforcement activity since Operation Pangea II in 2009, which reported shut down of 153 websites and 12 arrests/individuals under investigation [53].

UNODC has also taken an active role in the global fight against transnational organized crime involved in the trafficking of counterfeit medicines [54]. This includes partnership with the International Narcotics Control Board, which has specifically called upon governments to engage in enforcement against illicit online pharmacies [55]. This partnership also included an emphasis on enforcement and prevention of online pharmacy use of social media marketing to target youth and children, one of the few times there has been international recognition of the emerging threat of illicit eDTCA use in Medicine 2.0 technologies [55].

Despite Interpol and UNODC-led efforts, lack of a sustained and internationally agreed upon multilateral/multistakeholder mechanisms for proactive identification, prevention, and enforcement against illicit online pharmacies persists. Global coordination is limited, allowing these virtual criminal actors to remain active worldwide and continually create new illicit eDTCA and cyberpharmacies. Though limited in effectiveness, Operation Pangea represents a potentially successful model of partnership and collaboration among the wide range of stakeholders necessary to address this problem and can be built upon strategically for future efforts. Indeed, without multi-lateral/sector cooperation, it is simply impossible to target and disable all relevant technologies supporting illicit online pharmacies [20].

Other public private partnership models (“PPPs”) have also been tried as potential governance mechanisms beyond Interpol’s Operation Pangea. For example, the Alliance for Safe Online Pharmacies and Center for Safe Internet Pharmacies are attempting to coordinate efforts addressing marketing and sales by illicit online pharmacies in the USA [44,56,57]. In addition, countries such as China have also engaged in public-private collaboration, recently forming a partnership between Chinese search engine Baidu and the State Food and Drug Administration to provide online certification and search results identifying legitimate online pharmacies [58]. The effectiveness of these programs remains to be seen; however, without investment and tangible mechanisms to enable action and coordination across geopolitical lines, their effectiveness to address the complexity of the online pharmacy problem is questionable.

## Global health and internet governance

Our examination of governance efforts indicates that national government legislation, voluntary governance mechanisms, and global law enforcement efforts attempting to address the public health and cybersecurity risks of illicit online pharmacies have not been adequate, especially in the context of unregulated eDTCA. Illicit online pharmacy eDTCA use explicitly involves criminal actors that utilize false and misleading information meant to induce unjustified and unsafe use of medicines. These concerns clearly reside within the general principles of ethical standards enshrined in the WHO Criteria targeting industry-based promotion, yet which did not anticipate the development of illicit online pharmacies as a point of access and source of promotion. Further, even if the WHO Criteria had specifically addressed illegal online pharmacy promotion in its text, lack of adoption of its guidelines in national legislation would continue to limit effectiveness of its application. Improved global health and Internet governance is therefore urgently needed.

Illicit global trafficking of medicines via the Internet directly impacts individual patients and population-based health outcomes. Yet infrastructures enabling this illicit e-commerce are primarily IT and private sector driven [19,20]. Consequently, although a public health concern, combatting it must engage specialized partners to reflect the criminal nature of the perpetrators, global networks of conspirators, technical nature of the crime, and health harms that ensue from these illicit activities. An effective solution begins with enhanced and inclusive governance mechanisms engaging multidisciplinary actors from global public health, but also IT and law enforcement entities empowered to fight transnational organized forms of cybercrime. Leveraging existing Internet and health governance structures, raising awareness to this form of cybercrime, and creating a new paradigm for “e-Health Governance” can form the foundation for this strategy.

### Evolving internet governance

“Internet governance” is a relatively new phenomena. Conceptually, it is defined as the establishment of shared principles, norms, rules, decisionmaking procedures and programs developed by governments, the private sector, and civil society on the use and evolution of the Internet [59]. Reflecting a heretofore decentralized, multi-stakeholder, multi-country, interconnected, self-governed and autonomous group of actors, the UN has made Internet governance a global priority despite its highly challenging nature.

Beginning in 2005, the UN-initiated World Summit on the Information Society (“WSIS”) established the Internet Governance Forum (“IGF”) to engage, in an open and inclusive manner, multiple stakeholders in a policy dialogue regarding Internet governance [60]. Importantly, IGF led to inclusion of an expanding set of international Internet policy issues for debate. Originally, Internet governance focused narrowly on technical aspects (i.e., protocols, infrastructure) but now has evolved to include international policy development on issues such as security, stakeholder information exchange and engagement, and, crucially, finding solutions to issues arising from the misuse of the Internet [61].

IGF has been successful in engaging a wide array of stakeholders, including national governments, the private sector, civil society, academia, and other technical communities [61]. Importantly, these include public health, law enforcement, and Internet experts/groups such as ICANN, Interpol, ITU, WHO, the World Wide Web Consortium, Council of Europe (which has its own treaty, the MEDICRIME Convention, the first binding international instrument addressing the counterfeiting of medical products and similar crimes involving threats to public health from a criminal law standpoint [62]), Hewlett-Packard (which has developed its own mPedigree mobile medicines authentication system), and numerous others [63].

As a UN Summit, WSIS is relatively flexible, allowing primary agenda setting by UN Member States, broad engagement with other UN agencies, while intergovernmental organizations, accredited civil society and private sector entities, and other associated entities can participate as observers [60]. However, IGF’s structure is much more developed and inclusive, with a Multistakeholder Advisory Group comprised of members from national governments, civil society, the private sector, as well as academic and technical communities that provide information to the UN Secretary General on programmatic activities [64].

### A foundational proposal: e-health governance for cybercrime

Extant Internet governance approaches are very useful in addressing online health activities. WSIS and IGF’s structures and the WSIS plan of action have stated goals of building an “inclusive Information Society” promoting international and regional cooperation, incorporating public-private partnership (“PPP”) models into its action plans, promoting e-Health technologies and quality of online health information, and *expressly* noting the need to take appropriate measures to combat illegal and harmful media content [65].

Building upon emerging Internet governance, we believe an enhanced “e-Health Governance” model for cybercrime can be created, beginning a coordinated and focused effort to address illicit online pharmacies and their fraudulent and misleading use of eDTCA. Foundationally, this would entail promoting global health diplomacy efforts in all Internet governance activities, consistently prioritizing illicit online pharmacies as a preeminent cybersecurity and cybercrime issue, and building protections so that eDTCA is not false or misleading for consumers.

Proposed e-Health Governance for cybercrime should be shaped like the more inclusive IGF infrastructure and include its broad membership. This is both most acceptable and apt, as IGF stakeholders have already begun discussions regarding eDTCA regulation, counterfeit medicines in developing countries, pharmaceutical authentication technologies, and fraudulent commercial practices of illicit online pharmacies in the context of subjects regarding international trade, privacy and security, digital access, and improving Internet governance [66-68]. IGF is an extant, well-established, functional, and broadly competent group that can garner efficiencies as well as avoid limitations of existing governance mechanisms that fail to engage necessary and broader stakeholder participation [22].

However, beyond IGF infrastructure and membership, crucial to the success of e-Health cybercrime governance is partnership with organizations that currently focus on illicit online pharmacy networks, transnational crime, and cybersecurity. Here, the UNODC is well situated to coordinate IGF partner efforts. First, UNODC is the lead UN agency combatting global organized criminal networks, including trade in counterfeit medicines. Importantly, it has widespread political support and existing partnerships with organizations such as Interpol, the World Customs Organization and civil society that are already active in the fight against counterfeit medicines [22].

Second, UNODC is empowered by the UN Convention against Transnational Organized Crime (“UNTOC”) [22,69,70]. UNTOC allows UNODC to address serious global crimes, including human trafficking, smuggling, and illicit manufacture and trafficking of dangerous materials [69]. UNTOC also has near universal global adoption; 174 Member States are party to the Convention. Under UNTOC, UN Member States commit themselves to enact domestic laws against organized crime and collaborate internationally to fight against criminal networks.

UNODC and application to UNTOC have recently converged regarding illicit online pharmacy and fraudulent eDTCA cybercrime–focused issues. At the 2011 20^th^ Session of UN Commission on Crime Prevention and Criminal Justice (“CCPCJ”), three resolutions were adopted that clearly have reinforced global empowerment of UNODC to fight illicit online pharmacy activities: Resolution 20/4, “Promoting further cooperation in countering transnational organized crime,” Resolution 20/6, “Countering fraudulent medicines, in particular their trafficking” (“fraudulent” medicines defined by the CCPCJ as those whose contents are inert, expired, or otherwise different from what indicated,) and Resolution 20/7, “Promotion of activities relating to combating cybercrime, including technical assistance and capacity-building [71].” Each contemplates and calls for UNODC leadership based on its unique capabilities, empowerment, transnational experience and/or its successful, inclusive partnerships with other stakeholders.

Leveraging its support and empowerment, UNODC can engage IGF and WSIS stakeholders to promote e-Health Governance investigation, detection, and coordination activities against illicit online pharmacies and their misleading eDTCA. Further, a UNODC-IGF partnership infrastructure can spearhead additional legal and enforcement capacity by creating model national legislation to address criminal oversight of online pharmacies, particularly given the current absence of regulatory development in the vast majority of countries worldwide [15].

Once established, several matters could be on its early agenda. We believe five fundamental matters should be addressed as permanent agenda items in UNODC-led e-Health Governance efforts focusing on illicit online pharmacy related cybercrime. They focus upon security, diplomacy, partnerships, credentialing, and criminal surveillance strategies (see Table 1). Through this infrastructure and permanent agenda, dynamically adjusted as technology evolves, a UNODC-IGF e-Health Governance solution for cybercrime can begin the process of creating effective legal and technical barriers against illicit online pharmacies and their fraudulent eDTCA use.

**Table 1:** Fundamentals of UNODC-IGF e-health governance on cybercrime

**Area of focus**	**Description**	**Goals**	**Activities**
**e-Health security**	Dynamic Coalition Working Group (DCWG) in IGF comprised of stakeholders from public health, information technology and law enforcement communities.	Develop a “best practices” or similar agreed upon set of recommendations regarding Internet security and access specifically tailored to needs of global public health and regulating Internet pharmacies.	Development of a living document that should be revisited as experience in cybercrime grows in the health sector. The primary issue for this working group to address is ensuring patients with safe and quality access to health information and services online, including appropriately regulating eDTCA and Internet pharmacies.
**Global e-Health diplomacy**	Development of special Multistakeholder Advisory Group (MAG) of IGF with permanent membership that advises the UN Secretary General on issues of cybercrime and health.	UNODC-IGF MAG would raise awareness and engage in health diplomacy regarding currently unaddressed areas of transnational cybercrime involving health, specifically emphasizing cybercrime and public health risks of illicit online pharmacies.	MAG should participate in and advocate for e-Health Governance issues in future WSIS regional preparatory meetings, WSIS + 10 High Level Meeting in 2014, and Overall Review of the Implementation of WSIS Outcomes in 2015, focusing on the serious public health and cybersecurity concerns from illicit online pharmacies.
**Public-private partnership models**	Development of a structured mechanism to engage multiple public and private stakeholders to form public-private partnership (PPP) models addressing cybercrime and health in WSIS and IGF.	Creation and investment in PPP pilot projects specifically addressing cybercrime perpetrated by illicit online pharmacies. Active participation of UNODC, Interpol, WHO, the branded and generic pharmaceutical and wholesaler industries, the Internet service sector, patient safety and medical professional societies, as well as other stakeholders should be sought from onset.	PPPs in global health have come under scrutiny regarding the need for transparency and mitigating conflicts of interest. However, PPPs in Internet governance have generally not been subject to the same scrutiny as private sector participation is essential and necessary in the operation and maintenance of the Internet. Hence, PPPs in the Internet governance fora that focus on cybercrime can provide a sustained pathway for collective action/enforcement and continued investment.
**Health information credentialing for consumers**	DCWG or MAG will review and explore the merits of existing online credentialing systems for online pharmacies.	Development and deployment of a globally harmonized credentialing system that is easy for consumers to understand and use for purposes of purchasing medications online.	The NABP VIPPS program as well as proposed EU systems should be assessed and determination of a potential global standard considered. Use of other third party Internet surveillance companies should also be explored. Other alternatives that promote easy consumer verification of legitimate entities should also be explored such as creation of monitored and accredited generic top-level-domain names and investment in programs to increase digital health literacy.
**Cybercrime tools**	DCWG and PPPs jointly working together to develop technical capacity and necessary tools for cybercrime surveillance, prevention and enforcement against illicit online pharmacies.	Because of its unique technical expertise regarding Internet governance and transnational organized crime, UNODC-IGF should identify and incorporate current global IT surveillance, prevention, communication and enforcement strategies into effective tools against illicit online pharmacies.	Joint development of technologies to proactively detect and remove online content using web crawlers/spiders, anti-spam filters, IP blocking, suspension of electronic financial transactions/processing, domain name server monitoring and surveillance, fraud detection tools, as well as other strategies to combat illicit online pharmacies and their fraudulent eDTCA marketing.

## Conclusions

As illicit online pharmacies continue to proliferate and target patients globally with misleading and fraudulent forms of eDTCA, multistakeholder-based governance efforts must be created to effectively address this dangerous form of cyber and public health crime. The focus of any eHealth Governance approach must be on ensuring appropriate competencies and leadership are included, leveraging of resources, and the coordination and cooperation between the public health, IT, and the law enforcement international community. Using UNODC in combination with IGF provides such an opportunity. It is essential that the global community act collaboratively to address the unprecedented threat posed by illicit online pharmacies and their unregulated use of eDTCA. By promoting health and security in all forms of Internet governance, eHealth Governance systems can develop dynamically to ensure global patients are safe from dangerous, misrepresented medicines online today, tomorrow, and for future generations to come.

## Abbreviations

BRIC: Brazil-Russia-India-China (emerging markets); CCPCJ: The Commission on crime prevention and criminal justice; DTCA: Direct-to-consumer adverstising; DCWG: Dynamic coalition working group; eDTCA: Electronic or digital forms of direct-to-consumer advertising; FDA: United State of American food and drug administration; GOe: World Health Organization global observatory for e-health; ICANN: The internet corporation for assigned names and numbers; IGF: Internet governance forum; Interpol: International criminal police organization; IT: Information technology; ITU: International telecommunications Union; LMICs: Low-and-middle income countries; MAG: Multistakeholder advisory group; NABP: National association of boards of pharmacy; PPP: Public-private partnership; UNODC: United nations office of frugs and crime; WHO: World Health Organization; WHO Criteria: World Health Organization ethical criteria for medicinal drug promotion; WSIS: World summit on the information society.

## Competing interests

TKM (TKM) and BAL (BAL) received no extramural support from any organization for the submitted work that has an interest in the subject or that would present a conflict of interest. TKM is the 2011-2013 CLA Fellow of the Partnership for Safe Medicines (PSM), which supports his general research activities. BAL is a voluntary board member and Vice President of PSM, and receives no compensation for any PSM activities. PSM is not connected with the submitted work. BAL also serves as a member of the US Agency for Healthcare Research and Quality, Healthcare Safety and Quality Research Study Section, and the National Patient Safety Foundation Research Program Committee, both of which consider grant proposals addressing medication safety. TKM and BAL report no other relationships or activities that could appear to have influenced the submitted work.

## Authors’ contributions

We note that with respect to author contributions, TKM and BAL jointly conceived the study, TKM and BAL jointly wrote the manuscript, TKM, and BAL jointly edited the manuscript, and BAL supervised its legal and policy analysis. Both authors read and approved the final manuscript.
